# Growth, structural, thermal, and optical characteristics of L-asparagine monohydrate doped magnesium sulphate heptahydrate semiorganic crystals

**DOI:** 10.1016/j.heliyon.2023.e22322

**Published:** 2023-11-14

**Authors:** Md Anisur Rahman, Jiban Podder, Harinarayan Das

**Affiliations:** a Department of Physics, Bangladesh University of Engineering and Technology, Dhaka 1000, Bangladesh; b Department of Basic Sciences and Humanities (Physics), University of Asia Pacific, Dhaka 1209, Bangladesh; c Atomic Energy Center, Bangladesh Atomic Energy Commission, Dhaka, Bangladesh

**Keywords:** L-asparagine, MgSO_4_·7H_2_O, Structural properties, Thermal characterization, UV–Vis spectroscopy, Optical band gap energy

## Abstract

A novel semi-organic crystal has been grown using slow evaporation technique by doping organic compound L-asparagine monohydrate (C_4_H_8_N_2_O_3_·H_2_O) with inorganic material Magnesium sulphate heptahydrate (MgSO_4_·7H_2_O). The crystallographic parameters like strain, dislocation density and crystallite size were calculated by powder X-ray diffraction method. Functional groups were identified and bond length, force constants were calculated from FT-IR spectroscopy. Energy dispersive X-ray (EDX) analysis was used to identify the constituent elements of the crystal. Kinetic and thermodynamic parameters, such as, activation energy E_a_, change in Gibb's free energy (ΔG) and change in enthalpy (ΔH) have been determined by thermogravimetric analysis (TGA) analysis. E_a_, ΔH and ΔG show positive values and change in entropy (ΔS) shows negative ones. The thermal degradation behavior of the crystals has been analyzed by differential scanning calorimetry (DSC) analysis. Various optical constants such as optical band gap, lattice dielectric constant, absorbance, extinction coefficient, the ratio of free charge carrier concentration to the effective mass, Urbach energy, optical and electrical conductivities were estimated from UV–vis transmittance data. High optical conductivity (10^10^ s^−1^) justifies the good photo response nature of the semi-organic crystal.

## 1 Introduction

Frequency mixing, photonics, second harmonic generation and optoelectronics are the rapidly developing fields of modern era where the use of organic non-centrosymmetric compounds has played a pivotal role [1]. It is the π-bonds in the organic materials that cause high nonlinear susceptibility (χ) due to molecular hyperpolarizability (β). However, these organic crystals have some unfavorable features, such as volatility, low thermal stability and poor mechanical strength [2]. In comparison, inorganic materials have low nonlinear efficiency but excellent thermal and mechanical stability. Therefore, the growth of a new type of material known as semi-organic crystal has become a recent trend, where the strong physical and chemical features of inorganic crystals are combined with the excellent nonlinear optical characteristics of organic materials [3]. Over the past few decades, organic materials based on amino acid and their compounds have attained increasing significance for application in NLO devices. L-asparagine monohydrate is a well-known NLO substance in the amino acid group. Numerous studies have been conducted on L-asparagine doped compounds, notably l-asparagine l-tartaric acid, L-asparaginium picrate, and L-asparaginium L-tartrate [[4], [5], [6]]. Magnesium sulphate heptahydrate (MgSO_4_·7H_2_O) single crystal has become a significant compound of study for both academic and industrial purposes [7]. Accordingly, in the current study, single crystals of magnesium sulphate heptahydrate (MSHH) have been grown in pure form and semi-organic crystals have been grown by doping l-asparagine monohydrate (LAM) with MSHH using the slow evaporation method. The grown crystals have been characterized for thermal, structural, and optical properties.

## 2 Growth of the crystals

**Fig. 1:**
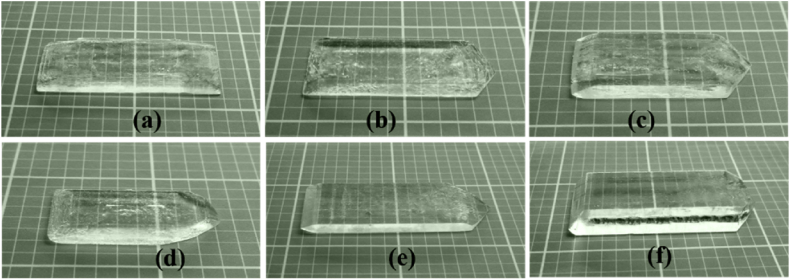
Image of the grown crystals: (a) MSHH + 0 mol% LAM, (b) MSHH + 0.2 mol% LAM, (c) MSHH + 0.4 mol% LAM, (d) MSHH + 0.6 mol% LAM, (e) MSHH + 0.8 mol% LAM, (f) MSHH + 1.0 mol% LAM.

The crystal was grown in pure form and by adding AR grade LAM with MSHH in double-distilled water. Later the solution was stirred constantly for 4 h with a magnetic stirrer, and finally, Whatmann filter paper was used to filter the solution. Recrystallization was carried out to improve the crystal's quality. Thereafter, the solution was poured into a beaker and covered by a transparent plastic sheet with small pores. Finally, it was placed in a dust-free chamber. After 40 days, transparent and well-shaped crystals were obtained as shown in Fig. 1.

## 3 Characterization method

The finely powdered crystals were subjected to powder X-ray diffraction test by Philips X'Pert PW 3040 Powder X-ray Diffractometer over the 2θ range of 10–80°, with angle step size of 0.02° with CuK_α1_ radiation (λ = 0.154 nm). The UV–Vis spectrophotometer (Shimadzu, UV-3100, Japan) was used to measure the optical transmittance spectrum of the crystals in the range of 190–1100 nm. For measuring the transmittance spectra, the crystal was gently polished until a thickness of 2 mm is reached. Simultaneous Thermal Analyzer STA 449 F^3^ Jupiter was used to perform the TG/DSC at a heating rate of 10 K/min in nitrogen atmosphere. The FTIR spectra of the powdered samples were recorded in the range of 4000–350 cm^−1^ using the KBr pellet technique by STA 449 F^3^ Jupiter.

## 4 Results and discussion

### 4.1 Identification of the crystal system of grown crystals

**Fig. 2:**
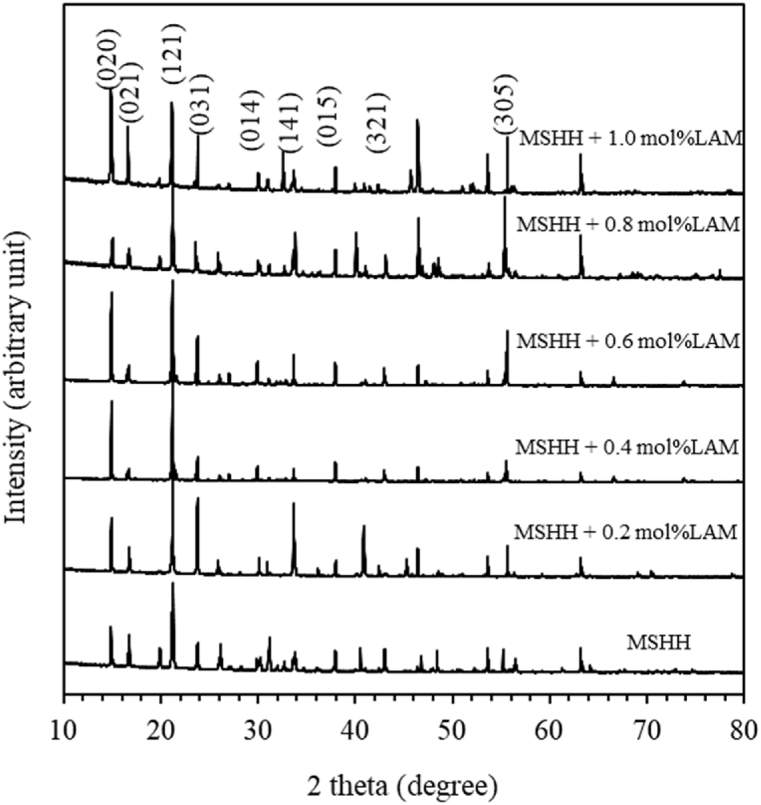
XRD pattern of pure and LAM doped MSHH crystal.

The narrow, strong and sharp peak of the X-ray diffraction is the evidence of growth of single crystal. The study identifies that the crystal belongs to orthorhombic nature with non-centro symmetric space group P_212121_ (Fig. 2). The lattice parameters of the unit cell were estimated from the d-spacing values provided by the instrument and the corresponding ESDs are also listed in Table 1. The unit cell volume is observed to increase from 948.890 (Å)^3^ to 969.893 (Å)^3^ indicating LAM incorporation into MSHH crystal structure. It is also seen that the calculated and the reported values match quite well.

**Table 1:** Lattice parameters and calculated ESDs of pure and LAM doped MSHH crystals.

Powder sample	a (Å)	ESD in a (Å)	b (Å)	ESD in b (Å)	c (Å)	ESD in c (Å)	Volume (Å)^3^
MSHH (JCPDS no. 96-900-7484)	6.857		11.868		11.996		976.221
MSHH +0 mol% LAM	6.773	0.063	11.911	0.030	11.761	0.074	948.890
MSHH +0.2 mol% LAM	6.525	0.028	12.309	0.031	11.827	0.003	949.980
MSHH +0.4 mol% LAM	6.544	0.039	12.331	0.044	11.877	0.045	958.368
MSHH +0.6 mol% LAM	6.537	0.029	12.337	0.042	11.930	0.029	962.076
MSHH +0.8 mol% LAM	6.769	0.029	11.934	0.047	11.929	0.028	963.683
MSHH +1.0 mol% LAM	6.796	0.079	12.136	0.034	11.759	0.017	969.893

#### 4.1.1 Determination of crystallite size, strain, and dislocation density

The crystallite size (d) has been obtained using Scherrer's Eq. (1).

(1) $$d=\frac{k\lambda }{\beta \;\cos\;\theta }$$

where the value of constant k is 0.9 (only for spherically shaped particle [8]), the wavelength of X-rays (λ) is 0.154 nm, and full width at half maximum (FWHM) of the peak is represented by β. The strain (*ε*) was determined from the slope of the curve, plotted between βcosθ and 4sinθ representing Williamson Hall Eq. (2).

(2) $$\beta \;\cos\;\theta =4\varepsilon \;\sin\;\theta +\frac{k\lambda }{d}$$

The dislocation density has been determined using Eq. (3) as follows:

(3) $$\delta =\frac{1}{d^{2}}$$

Crystallite size, strain, dislocation density are given in Table 2. The crystallite size is observed to increase from 99.039 nm to 154.060 nm and strain from 0.3 to 1.4, which reveals incorporation of LAM into MSHH crystal matrix. The dislocation density is seen to decrease from 0.1020 × 10^−3^ nm^−2^ to 0.0421 × 10^−3^ nm^−2^ due to the addition of LAM, which is a clear indication of good crystallinity.

**Table 2:** Crystallite size, strain, dislocation density of pure and LAM doped MSHH crystals.

Powder sample	Crystallite size D (nm)	Strain *ε* ( × 10^−3^)	Dislocation density δ ( × 10^−3^) (nm^−2^)
MSHH + 0 mol% LAM	99.039	0.3	0.1020
MSHH + 0.2 mol% LAM	106.657	0.4	0.0879
MSHH + 0.4 mol% LAM	115.545	0.1	0.0749
MSHH + 0.6 mol% LAM	126.049	0.6	0.0629
MSHH + 0.8 mol% LAM	138.654	0.7	0.0520
MSHH + 1 mol% LAM	154.060	1.4	0.0421

### 4.2 FTIR spectroscopy analysis

**Fig. 3:**
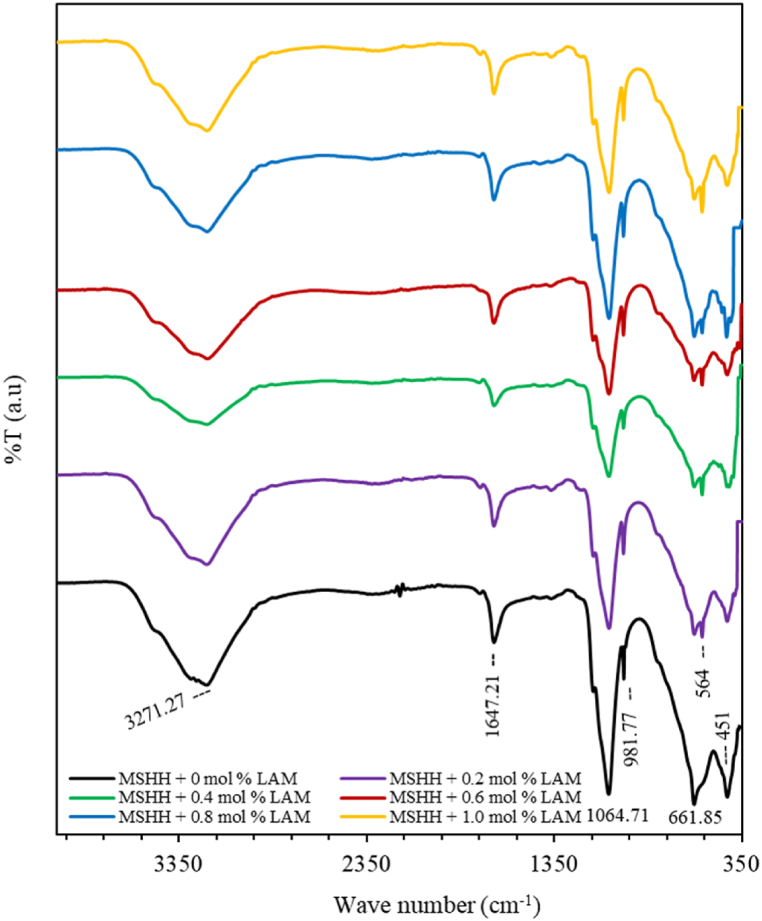
FTIR spectra of pure and LAM doped MSHH crystal.

The FTIR spectra of a pure and LAM-doped MSHH crystal are shown in Fig. 3. The broad band at 3271.27 cm^−1^ with the characteristic OH stretching is caused by water molecules. At 1647.21 cm^−1^, the HOH bending vibrational band is observed. The peak for SO_4_^−2^ bending vibration can be seen at 981.77 cm^−1^, while the peak at 1064.71 cm^−1^ is the absorption band caused by SO_4_^−2^ stretching vibration. MgO group is reported to cause the peak at 661.85 cm^−1^ [9]. At 451 cm^−1^, the T_d_ symmetry of the SO^−2^ ion can be seen [10]. The additional peak in spectra of LAM doped MSHH crystals at 564 cm^−1^ is caused by torsional oscillation of NH_3_^+^, which confirms the presence of LAM content in the doped sample.

Eq. (4) is used to calculate the bond length of the functional groups. The unit of the force constant k is measured in dynes per centimeter.

(4) $$r=\sqrt[{3}]{\frac{17}{k}}$$

Hook's law is applied in eq. (5) to determine the force constant (k) which is an indicator of the stiffness of the bond [11]. Bonds having shorter lengths are thought to be stronger and have a greater force constant (k).

(5) $$\nu =\frac{1}{2\pi c}\sqrt{\frac{k}{\mu }}$$

where μ is the reduced mass (g) of the atoms being bonded and derived using Eq. (6) [12]:

(6) $$\mu =\frac{m_{1}.m_{2}}{m_{1}+m_{2}}$$

where the atomic masses of the atoms are m_1_ and m_2_. Table 3 lists bond lengths, force constants of pure and LAM doped MSHH crystals.

**Table 3:** Bonds and their lengths, force constants of pure and LAM doped MSHH crystals.

Sl. No.	Bond	FTIR peaks (cm^−1^)	Force constant k (Mega dyne/cm)	Bond length r (Å)
1	OH	3271.27	0.5938	3.0591
2	HOH	1647.21	0.1506	4.8333
3	SO_4_^−2^	1064.71	0.7129	2.8783
4	SO_4_^−2^	981.77	0.6062	3.0382
5	MgO	661.85	0.2479	4.0930
6	SO_4_^−2^	451	0.1279	5.1032
7	NH_3_^+^	564	0.0175	9.9031

### 4.3 Energy dispersive spectral analysis

**Fig. 4:**
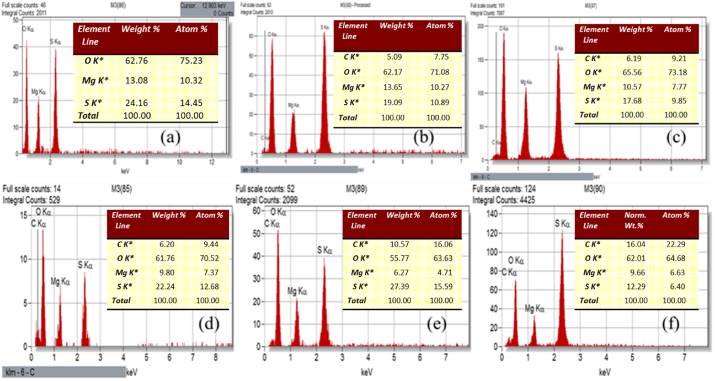
EDX spectra of pure and LAM doped MSHH crystal, (a) pure MSHH, (b) MSHH + 0.2 mol % LAM, (c) MSHH + 0.4 mol % LAM, (d) MSHH + 0.6 mol % LAM, (e) MSHH + 0.8 mol % LAM, (f) MSHH + 1.0 mol % LAM.

Energy dispersive X-ray spectra shown in Fig. 4 provide evidence that LAM molecule has been absorbed into MSHH crystal structure. Additionally, the stoichiometries distribution of the chemical composition in the pure and LAM doped MSHH crystal is validated by the use of EDX spectra. The carbon content of LAM is observed to increase with doping concentration (Fig. 4).

### 4.4 Thermal analysis

**Fig. 5:**
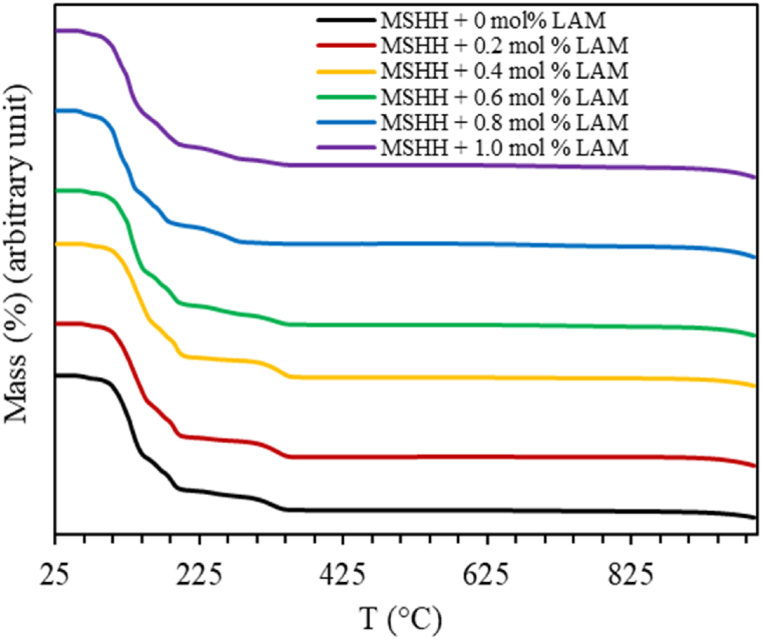
TGA curve of pure and LAM doped MSHH.

**Fig. 6:**
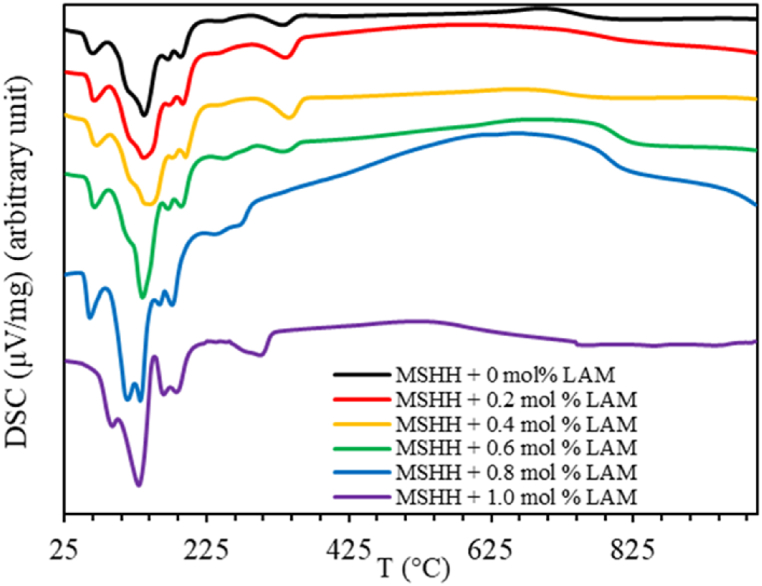
DSC curve of pure and LAM doped MSHH.

Thermal analysis of the grown crystals has been conducted using thermogravimetric (TG) and differential scanning calorimetric (DSC) techniques. While DSC calculates heat (enthalpy) changes with temperature or time and TG analysis shows the mass change with the same [13]. Numerous kinetic and thermodynamic parameters are computed from TG curves. The TG profile in Fig. 5 shows that the mass loss happens in five stages and a respective shift in DSC curve is also evident. The first stage occurs in 60–80 °C temperature range where 1.5 % mass loss is observed, which indicates the release of one water molecule. According to Fig. 6, an endothermic reaction occurs in the initial dehydration stage, which is confirmed by the negative DSC peak [14].

The weight loss of 30 % appears as a steep fall in the second stage with a notable peak between 82 °C and 145 °C. A loss of roughly four water molecules of MSHH crystal and five water molecules for LAM doped MSHH samples may be responsible for the rapid decrease in mass [14,15]. The third stage, which occurred between 145 and 203 °C, represents 12 % of mass loss showing a steady decrease in weight loss. In total, six water molecules are lost in the second and third phases representing a mass loss of about 42 % [14]. An endothermic transition is evident by the negative DSC peak in the second and third dehydration steps. The fourth stage occurs between 204 and 221 °C. Between 291 and 365 °C, fifth stage is observed displaying a rate of 5 % mass loss which is slower than the previous stages. These phases feature the decomposition of MSHH molecules.

This indicates that MgSO_4_·6H_2_O is changed to anhydrous MgSO_4_ during these fourth dehydration steps [14]. It is believed that successive decompositions are driven by the removal of SO_4_^−2^ ions, which changes into SO_2_ and O_2_ and finally leave MgO as residue [10].

MgSO_4_·7H_2_O → MgSO_4_ + 7H_2_O

MgSO_4_ → MgO + SO_2_↑ + O_2_↑

In Fig. 6, all DSC curves are observed to have endothermic peaks below 230 °C, confirming the dehydration step where energy is needed to eliminate moisture. The degradation of the crystal is indicated by succeeding exothermic peaks. Phase change is attributed to the small peak at about 340 °C.

#### 4.4.1 Calculating the rate constant and half-life of the reaction by the TGA graph

Eq. (7) is a first-order reaction rate equation that has been applied to derive the activation energy along with other thermodynamic parameters [16].

(7) $$\frac{dx}{dt}=k\left(1-x\right)$$

Where, $=\frac{w_{i}-w_{t}}{w_{i}-w_{f}}$ , *w*_*t*_ is the sample's weight at a specific time *t*, *w*_*i*_ and *w*_*f*_ are starting and final weights. From Eq. (7), we can write

(8) ln(1−x)=−kt

If a graph is plotted using Eq. (8), then it can be seen that each phase traces a straight line, indicating that the changes are reactions of the first order. The slope of every line was used to calculate the rate constant (*k*) for a specific phase, and Eq. (9) was applied to calculate the half-life (*t*_1/2_). Table 4 provides the k and *t*_1/2_ values.

(9) $$t_{1/2}=\frac{0.693}{k}$$

**Table 4:** Kinetic and thermodynamic parameters of all phases during thermogravimetric analysis.

Parameter	Stages	MSHH +0 mol% LAM	MSHH +0.2 mol% LAM	MSHH +0.4 mol% LAM	MSHH +0.6 mol% LAM	MSHH +0.8 mol% LAM	MSHH +1.0 mol% LAM
Rate constant k (min^−1^)	Dehydrartion (1st stage)	0.1906	0.1851	0.0662	0.1637	0.1919	0.1111
	Dehydrartion (2nd stage)	0.041	0.0255	0.0041	0.0551	0.0604	0.2314
	Dehydrartion (3rd stage)	0.2234	0.1826	0.0213	0.1358	0.1091	0.3245
	Dehydrartion (4th stage)	0.0051	0.0023	0.0003	0.0053	0.0043	0.3672
	Decomposition (5th stage)	0.032	0.045	0.056	0.041	0.024	0.101
Half life T_1/2_ (min)	Dehydrartion (1st stage)	3.636	3.744	10.468	4.233	3.611	6.238
	Dehydrartion (2nd stage)	16.902	27.176	169.024	12.577	11.474	2.995
	Dehydrartion (3rd stage)	3.102	3.795	32.535	5.103	6.352	2.136
	Dehydrartion (4th stage)	135.882	301.304	2310.000	130.755	161.163	1.887
	Decomposition (5th stage)	21.656	15.400	12.375	16.902	28.875	6.861
ΔH (Jmol^−1^) ( × 10^3^)	Dehydrartion (1st stage)	118.10	84.42	76.65	67.34	66.89	7.67
	Dehydrartion (2nd stage)	74.90	76.29	60.94	65.43	67.79	7.57
	Dehydrartion (3rd stage)	15.38	16.81	10.20	17.07	18.02	15.1
	Dehydrartion (4th stage)	30.62	30.41	9.12	10.65	9.73	113.73
	Decomposition (5th stage)	3.62	6.29	10.14	10.46	112.55	76.64
ΔG (Jmol^−1^) ( × 10^4^)	Dehydrartion (1st stage)	24.22	23.22	26.36	25.49	31.26	13.53
	Dehydrartion (2nd stage)	22.72	24.53	25.71	32.13	17.19	15.95
	Dehydrartion (3rd stage)	25.64	26.19	36.17	15.89	15.88	14.36
	Dehydrartion (4th stage)	25.93	37.61	17.86	17.68	16.53	944.70
	Decomposition (5th stage)	36.14	15.62	16.41	14.23	941.24	680.68

**Fig. 7:**
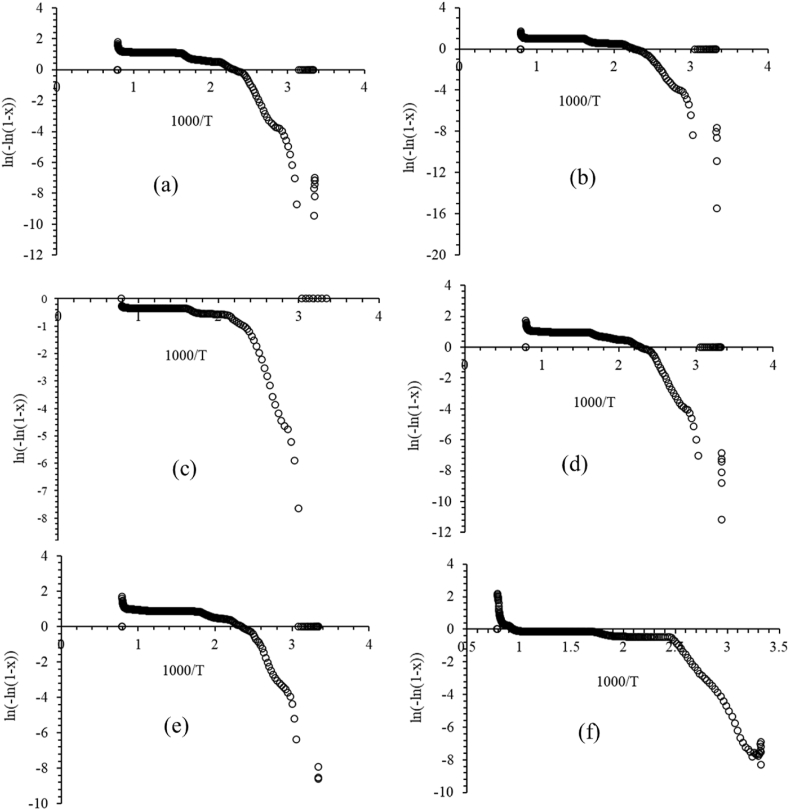
Plot of ln [-ln (1 - x)] vs 1000/T of pure and doped MSHH crystals. (a) Pure MSHH, (b) MSHH + 0.2 mol % LAM, (c) MSHH + 0.4 mol % LAM, (d) MSHH + 0.6 mol % LAM, (e) MSHH + 0.8 mol % LAM, (f) MSHH + 1.0 mol % LAM.

The modified Coats and Redfern model is represented by equation (10). The kinetic parameters have been determined from this equation [[17], [18], [19], [20], [21], [22], [23]]:

(10) $$\ln\left[-\ln\left(1-x\right)\right]=\ln\frac{ART^{2}}{\beta E_{a}}-\frac{E_{a}}{RT}$$

where E_a_ stands for activation energy, T indicates temperature in Kelvin, R is the gas constant (8.3143 J mol^-1^ K^−1^), A is the pre-exponential factor, and the heating rate β is 20 °C/min. The basic thermodynamic equations [24] were applied to calculate the remaining parameters. The activation energy for each phase is measured using graphs that are plotted with ln [ln (1 - x)] as y-axis and 1000/T as x-axis (Fig. 7). According to the values in Table 4, every phase indicates non-spontaneous endothermic process.

#### 4.4.2 Estimation of thermodynamic parameters

Eqs. (11), (12), (13) were used to calculate different thermodynamic parameters for each phase, including change in enthalpy (ΔH), change in Gibbs free energy (ΔG), and change in entropy (ΔS).

(11) $$\Delta H=E_{a}-RT$$

**Fig. 8:**
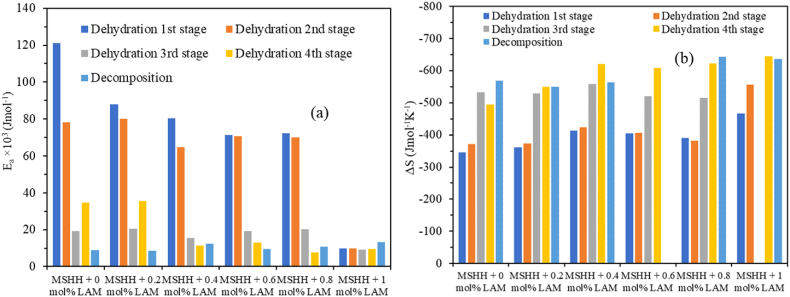
Plot of (a) activation energy and (b) change in entropy of various stages with doping concentration.

(12) ΔG=ΔH−TΔS

(13) $$\Delta S=k\left(\frac{Ah}{Tk_{b}}\right)$$

where A is the Arrhenius constant and k_b_ is the Boltzmann constant [24]. Table 4 lists the estimated values for the thermodynamic parameters k, t_1/2_, ΔH, ΔG, and E_a_, ΔS are displayed in Fig. 8. The change in enthalpy (ΔH) in a chemical reaction symbolizes the energy change between reactants and products. It has been found that there is a very low energy barrier (∼3 kJ/mol) between activation energy and change in enthalpy, indicating that the chemical reaction may begin easily [25].

### 4.5 Linear optical studies and relevant constants

Since the optical quality of a crystal is closely related to its electrical properties, atomic structure, and electronic band structure, it is very essential to know the optical characteristics of the crystal. In order to understand the optical behavior of a crystal, it is required to measure its optical transparency, absorption coefficient, extinction coefficient, band gap, and refractive index.

#### 4.5.1 Optical band gap

**Fig. 9:**
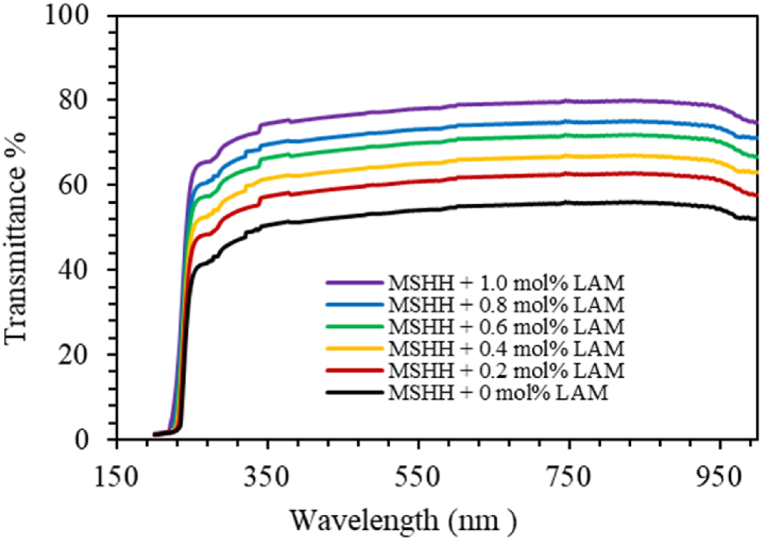
UV–Vis spectra of pure and doped MSHH crystals.

**Fig. 10:**
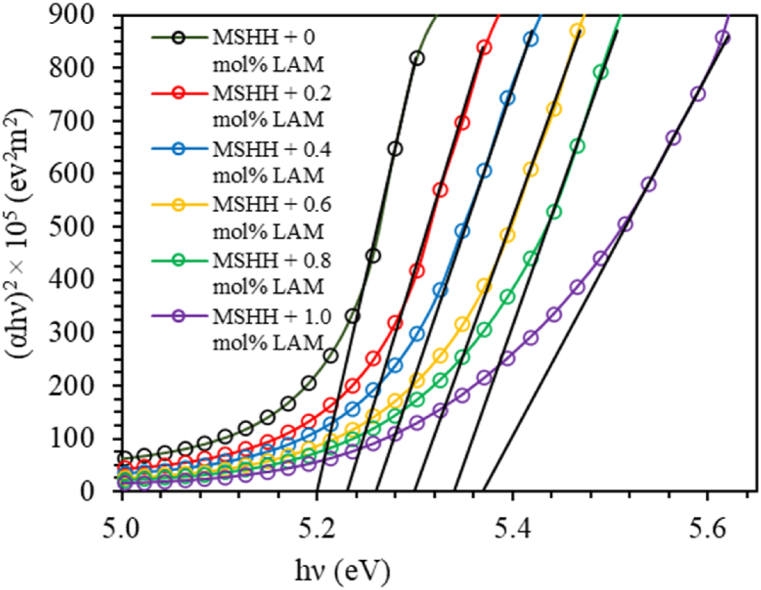
(*αhν*)^2^ as a function of photon energy for pure and LAM doped LSHH crystals.

Fig. 9 displays the optical transmittance of the grown crystals. Around at 257 nm, the cut-off wavelength is detected. The amine group having low scattering and low absorption nature is believed to eliminate defect centers making the doped crystals to have higher optical transmittance [26,27]. The absorption coefficient (α) was calculated by applying the equation:

(14) $$\alpha =\frac{2.303}{t}\times \log\left(\frac{100}{\%T}\right)$$

where t and T represent the sample's thickness and transmittance (%). The relation between photon energy (hυ) and absorption coefficient (α) is given by

(15) $$\alpha =\frac{A{\left(h\nu -E_{g}\right)}^{n}}{h\nu }$$

where A is a constant and E_g_ is the optical band gap [[28], [29], [30]], n is 1/2 and 2 for direct band gap and indirect band gap respectively. Table 5 lists the direct optical band gaps E_gd_ which are calculated from Tauc's plot (Fig. 10). An important attribute for photonics and optoelectronic devices is good dielectric nature, which is clearly indicated by an increase in band gap values of the doped crystals. Likewise, the doped crystal can be employed in solar panels as an antireflection coating that requires low reflectance, good band gap, low absorption, and high transmission. In a different way, the material's optical band gap E_th_ was also assessed by applying Plank's equation given below.

(16) $$E_{th}=\frac{hc}{\lambda_{\max}\left(nm\right)}$$

where λ is cut-off wavelength. The values of E_th_ matched reasonably with that E_gd_ as shown in Table 5. The high transmittance in the visible range is confirmed by the crystal's large band gap, and this crystal may be ideal for optoelectronic devices such as laser diodes [31].

**Table 5:** Optical parameters of pure and LAM doped MSHH crystals.

Optical Parameters	MSHH +0 mol% LAM	MSHH +0.2 mol% LAM	MSHH +0.4 mol% LAM	MSHH +0.6 mol% LAM	MSHH +0.8 mol% LAM	MSHH +1.0 mol% LAM
E_gd_ (eV)	5.2	5.23	5.26	5.3	5.34	5.37
E_th_ (eV)	5.27	5.31	5.34	5.36	5.36	5.38
ε_L_	5.0867	3.817	3.2726	2.723	2.4481	2.0618
N/m* (kg^−1^m^−3^)	4.41 × 10^55^	2.33 × 10^55^	1.65 × 10^55^	1.11 × 10^55^	8.75 × 10^54^	6.05 × 10^54^
E_u_ (eV)	0.24	0.23	0.22	0.21	0.2	0.19

#### 4.5.2 Extinction coefficient

**Fig. 11:**
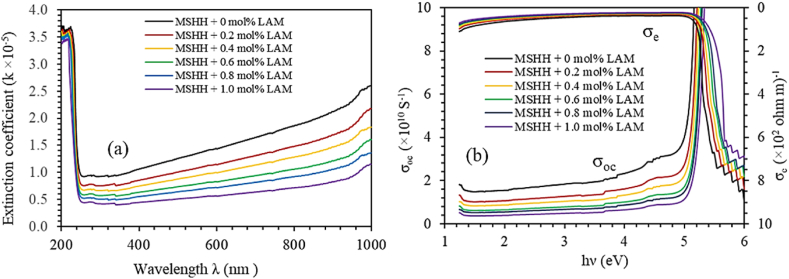
(a) Extinction coefficient of pure and doped MSHH crystals. (b) Relations of Electrical conductivity (σ_e_) and Optical conductivity (σ_o_) with photon energy for pure and LAM doped MSHH crystals.

The extinction coefficient estimates the amplitude decay of the incident magnetic and electric fields which is represented by the following equation [32].

(17) $$k=\frac{\alpha \lambda }{4\pi }$$

where the wavelength of the incident beam is λ. Fig. 11 depicts how the extinction coefficient changes with wavelength. It is observed that the extinction coefficient (k) increases with wavelength until the cut-off wavelength is met. The lower value of k is thought to be caused by a weak interaction between the electrons and photons in the material.

#### 4.5.3 Optical and electrical conductivity

The frequency response of the crystal to incident light, also known as optical conductivity (σ_op_) was determined by applying the equation shown in Ref. [33].

(18) $$\sigma_{op}=\frac{\alpha nc}{4\pi }$$

where c is the speed of light, n is the refractive index, and α is the absorption coefficient. Fig. 11 shows how optical conductivity changes with photon energy of the incident light. The optical conductivity is observed to reduce with the increase in molar concentration. The high photo-responsive behavior of the crystal is confirmed by a higher optical conductivity value (10^10^ s^−1^), which validates as an appropriate material for use in computing and information processing [34]. The transit of a large quantity of charge carriers from the valence to the conduction band is indicated by exponential growth at high energy values. A sudden increase in optical conductivity is observed at 5.2 eV confirming the exactness of energy band gap estimations. The electrical conductivity (σ_e_) can be written in terms of the optical conductivity and absorption coefficient as [35].

(19) $$\sigma_{e}=\frac{2\lambda \sigma_{op}}{\alpha }$$

Variations of electrical conductivity with photon energy are presented in Fig. 11. The lower range of electrical conductivity values reveals the dielectric nature of the crystal.

#### 4.5.4 Complex dielectric constant

Complex dielectric constant (ε_c_) can be determined by the following equation [36].

(20) $$\varepsilon_{c}=\varepsilon_{r}+i\varepsilon_{i}={\left(n+ik\right)}^{2}$$

where the real and imaginary parts of the dielectric constant are ε_r_ and ε_i_ respectively and their relation with the refractive index *n* and extinction coefficient *k* are as follows [37].

(21) $$\epsilon_{r}=n^{2}-k^{2}\text{and}\;\epsilon_{i}=2nk$$

**Fig. 12:**
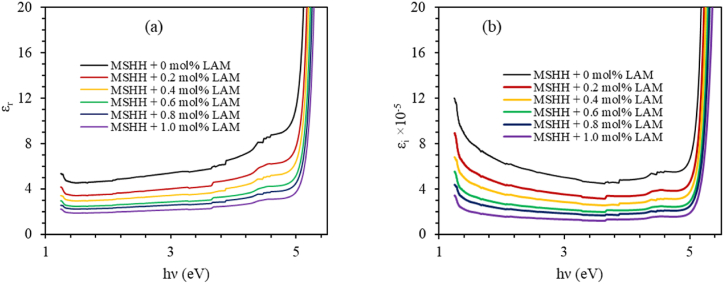
(a) Real and (b) imaginary part of dielectric constant with photon energy for pure and LAM doped MSHH crystal.

The parameters ε_r_ and ε_i_ are depicted as a function of photon energy in Fig. 12. The low value of the imaginary part of the dielectric constant is a crucial condition for optoelectronic device applications because it shows an impediment in the transmission of electromagnetic (EM) energy.

#### 4.5.5 Lattice dielectric constant and carrier concentration

**Fig. 13:**
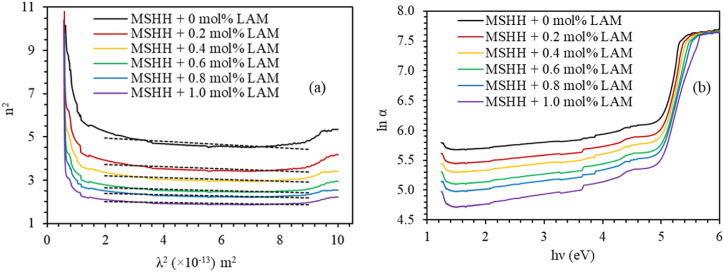
Plot of (a) n^2^ versus λ^2^, (b) ln α vs photon energy for pure and LAM doped MSHH crystals.

The following equation can be used to determine the lattice dielectric constant from the refractive index (n) [38].

(22) $$n^{2}=\varepsilon_{L}-\frac{e^{2}}{4\pi^{2}\varepsilon_{0}c^{2}}\frac{N}{m^{*}}\lambda^{2}$$

where e is the electronic charge, c is the speed of light, and (N/m*) is the ratio of the carrier concentration to the effective mass. The plot of n^2^ versus λ^2^, is shown in Fig. 13 (a). From the slope of the linear part and from the intercept of the straight line to the n^2^-axis, the lattice dielectric constant ε_L_ and ratio N/m × are calculated respectively and shown in Table 5. Table 5 also lists Urbach energy E_u_, which is determined from the plot of lnα versus photon energy as shown in Fig. 13 (b). The Urbach energy is found to decrease due to LAM doping, and this fall of Urbach energy is a clear indication of the decrease of defects in the doped crystals [39,40].

## 5 Conclusions

LAM doped MSHH semi-organic crystals with excellent optical quality have been grown by slow evaporation process from aqueous solutions and compared its properties with pure MSHH crystal. The XRD data showed that the grown crystals were orthorhombic. Additionally, the strong, sharp peaks and decrease of dislocation density show that LAM doped MSHH crystals have excellent crystalline structure. Increase of the unit cell volume, crystallite size and strain indicate the incorporation of LAM into MSHH crystal structure. Thermal investigation using TG and DSC showed that the doped crystals had improved thermal stability. All thermodynamic parameters, including a, ΔH, and ΔG values, are clearly positive while ΔS values are clearly negative, specifying a non-spontaneous chemical process. The fluctuation in activation energy is indicative that multi-reaction mechanisms exist during the process. It is evident from the DSC study that both endothermic and exothermic chemical processes are involved in the degradation process. The entrance of LAM molecule into the MSHH crystal lattice is confirmed by the energy dispersive X-ray and Fourier transform infrared spectroscopy. Furthermore, EDX analysis indicates the stoichiometric distribution of the C, Mg, S, and O components in the semi-organic crystal. LAM-doped MSHH semi-organic crystals have excellent transparency throughout the visible spectrum and can be applied to photonic and electro-optic devices. The band gap was determined based on UV–visible spectroscopy data and was found to increase with increasing doping concentration, making it suitable for applications in laser diodes. The estimated extinction coefficient confirmed its suitability for use in optical devices. For applications in optoelectronic devices, the low value of the imaginary part of the dielectric constant is a crucial requirement. The decrease in the Urbach energy and the increase in the optical band gap indicate that the defect bands have been partially eliminated due to doping. The large optical conductivity value indicates that the crystal has good photo responsive behavior, making it suitable for information processing and computer technology.

## Funding

This research did not receive any specific grant from funding agencies in the public, commercial, or not-for-profit sectors.

## Data statement

The data used for this article is derived from the experiments. These are not available online.

## Author's contributions

.

## Additional information

No additional information is available for this paper.

## CRediT authorship contribution statement

**Md Anisur Rahman:** Data curation, Formal analysis, Investigation, Software, Validation, Writing – original draft. **Jiban Podder:** Conceptualization, Data curation, Project administration, Resources, Supervision, Validation, Writing – review & editing. **Harinarayan Das:** Formal analysis, Investigation, Resources, Validation.

## Declaration of competing interest

The authors declare that they have no known competing financial interests or personal relationships that could have appeared to influence the work reported in this paper.
